# Weak Knees

**Published:** 1891-06

**Authors:** R. A. Chudleigh


### WEAK KNEES.

See if there be any bony displacement at the ankle or in the foot, or if there be a tendency to tread on the inner margin of the foot instead of flat on the sole. It is not likely that the mischief is located solely or chiefly in the knees. As the bones implicated are rapidly consolidating at the age of sixteen, no time should be lost in getting them into their normal curves; and how to effect this is not easily learnt by correspondence. An attempt at self-treatment may, perhaps, be made by placing two splints cased in wool, and reaching from the foot to considerably above the knee, one on the inside of the leg and the other on the outside, and bandaging the whole just tight enough not to be painful. This should be worn at night. If it prove too inconvenient to be worn by day also, shorter splints may be used; or perhaps by a little ingenuity a kind of hinge might be made at the knee, so as to admit of a little motion in the way of bending, while still stiff enough to give lateral support. It may also be advisable to impart a slope to the soles of the boots. But as it is not an easy thing to do all this by the light of nature, I would advise the farm laborer to get a ticket for advice at some dispensary or hospital, or else consult the local doctor.—R. A. CHUDLEIGH, *Wimborne.*

